# Venetoclax and hypomethylating agents versus tagraxofusp in older patients with blastic plasmacytoid dendritic cell neoplasm

**DOI:** 10.1007/s00277-025-06221-4

**Published:** 2025-02-20

**Authors:** Benjamin J. Lee

**Affiliations:** 1 Department of Pharmacy, Chao Family Comprehensive Cancer Center, University of California Irvine Health, 101 The City Drive South, Bldg. 23, Rm 275, Orange, CA 92868 USA; 2 Department of Clinical Pharmacy Practice, School of Pharmacy & Pharmaceutical Sciences, University of California, Irvine, CA USA

**Keywords:** BPDCN, Blastic plasmacytoid dendritic cell neoplasm, Venetoclax, Tagraxofusp

## Abstract

Blastic plasmacytoid dendritic cell neoplasm (BPDCN) is a highly aggressive and rare hematologic malignancy characterized by poor response to multiagent chemotherapy and dismal survival outcomes of 8–16 months. Tagraxofusp, the first-in-class CD123-directed antineoplastic agent, has emerged as a highly effective therapy and is the only FDA approved drug for BPDCN. Nonetheless, significant treatment-related toxicities with tagraxofusp such as hepatotoxicity and capillary leak syndrome are unfortunately not uncommon and can be prohibitive for older or unfit patients. The success of venetoclax (VEN) with a hypomethylating agent (HMA) has recently been described in the literature however, clinical outcomes are limited to case reports and small case series. To confirm these findings, we performed a multicenter, retrospective cohort study utilizing the TriNetX Networks database to compare survival outcomes between BPDCN patients (≥60 years-of-age) who received VEN + HMA versus tagraxofusp. In total, 32 and 39 patients received VEN + HMA and tagraxofusp, respectively, between February 1, 2019 and September 1, 2024. Median follow-up time was 7.4 and 9.3 months in the VEN + HMA and tagraxofusp cohorts, respectively. Overall survival (OS) between VEN + HMA and tagraxofusp-treated patients was comparable at 12-months (41.2% vs. 53%; HR 1.15; 95% CI, 0.53–2.48; *P* = 0.73). In a subgroup analysis of older adult patients (≥75 years-of-age), OS at 12-months (38.1% vs. 56.5%; HR 1.20; 95% CI, 0.47–3.04; *P* = 0.71) was not significantly different. In conclusion, this large-scale, retrospective database analysis suggests that VEN + HMA is an effective therapeutic alternative to tagraxofusp in older patients for the management of BPDCN. Future studies are needed to prospectively validate these findings.

**Supplementary Information:**

The online version contains supplementary material available at 10.1007/s00277-025-06221-4.

Blastic plasmacytoid dendritic cell neoplasm (BPDCN) is a rare, aggressive hematologic malignancy that commonly presents with multiorgan involvement of the skin, bone marrow, lymph nodes, and central nervous system (CNS). Historically associated with poor outcomes, response to multiagent cytotoxicity chemotherapy is reported in up to roughly 50% of treated patients and survival outcomes are dismal, ranging from 8 to 16 months [1–3]. The advent of tagraxofusp, a CD123 targeted antibody drug conjugate, has drastically changed the treatment paradigm for patients with BPDCN, which garnered FDA approval in December 2018. Despite tagraxofusp's efficacy in significantly reducing disease burden, its utilization carries risk for serious treatment-related toxicities.

Pre-clinical studies demonstrating high dependence of BPDCN on BCL-2 antiapoptotic function for survival have incited the adoption of venetoclax-based therapies for the management of older/unfit patients or those with relapsed/refractory disease [4–6]. While the combination of venetoclax and a hypomethylating agent (VEN + HMA) appears to have promising therapeutic potential for the treatment of BPDCN, clinical findings have been limited to case reports and small case series [5, 6]. To corroborate these findings, we performed a multicenter, retrospective cohort study utilizing the TriNetX platform, a global federated health research network that provides access to real-time, real-world electronic health records in an aggregate and de-identified form [7]. We evaluated older adult patients (≥60 years of age) with a diagnosis of BPDCN who received treatment with VEN + HMA or tagraxofusp between February 1, 2019 and September 1, 2024. To characterize baseline comorbidities of included patients, we reported composite ICD-10 code classifications for end organ impairment (Supplemental Table 1). Continuous variables were evaluated using the Student’s t-test and categorical variables were evaluated using the chi-square test, as made available through the TriNetX platform. The primary outcome of interest was overall survival (OS), which was compared between treatment groups using a 2-sided log-rank test. Hazard ratios (HR) were generated utilizing Cox-proportional hazard regression. All statistical analyses were conducted utilizing the TriNetX Analytics platform.

The TriNetX database was queried on November 17, 2024, and a total of 32 and 39 patients who received VEN + HMA and tagraxofusp, respectively, were identified from 22 healthcare organizations. The mean age was 75 years and 81.7% (*n* = 58) were male. History of acute or chronic kidney impairment was reported in 47.9% of patients while 62% had some form of cardiovascular disease or prior complication. Baseline comorbidities were comparable between VEN + HMA and tagraxofusp treated patients (Table 1). Median follow-up time was 7.4 months in the VEN + HMA cohort and 9.3 months in the tagraxofusp cohort. OS between VEN + HMA and tagraxofusp-treated patients was comparable at 12-months (41.2% vs. 53%; HR 1.15; 95% CI, 0.53–2.48; *P* = 0.73) (Fig. 1A). When a subgroup analysis was performed among older adult patients (≥75 years of age), OS at 12-months (38.1% vs. 56.5%; HR 1.20; 95% CI, 0.47–3.04; *P* = 0.71) was not drastically different (Fig. 1B).

**Table 1:** Baseline demographics and comorbidities

Variable ^a^	VEN + HMA (*n* = 32)	Tagraxofusp (*n* = 39)	*P* Value
Male, No. (%)	24 (75)	34 (87.2)	0.19
Age, (years)	75 ± 8	75 ± 7	0.84
BMI, (kg/m^2^)	29.1 ± 6.7	27.4 ± 6.0	0.34
History of kidney disease,^b^ No. (%)	19 (59.4)	15 (38.5)	0.09
History of liver disease,^c^ No. (%)	NR^f^	NR^f^	-
History of respiratory disease,^d^ No. (%)	22 (68.8)	19 (48.7)	0.09
History of cardiovascular disease,^e^ No. (%)	23 (71.9)	21 (53.8)	0.12

^a^ Reported as mean ± SD unless otherwise stated

^b^ Indicated by composite ICD-10 code for acute kidney failure, chronic kidney disease, and unspecified kidney disease (N17-N19)

^c^ Indicated by composite ICD-10 code for liver disease, including fibrosis and cirrhosis of the liver, inflammatory liver diseases, and other diseases of the liver (K70-K77)

^d^ Indicated by composite ICD-10 code for diseases of the respiratory system, including acute upper respiratory infections, influenza and pneumonia, chronic lower respiratory diseases, other diseases of the respiratory system (J00-J99)

^e^ Indicated by composite ICD-10 code for heart disease, including atrial fibrillation and flutter, heart failure, cardiomyopathy, and nonrheumatic valve disorders (I30-I5A)

^f^ Queries resulting in less than 10 findings are not reported by the TriNetX

Abbreviations: BMI, body mass index; HMA, hypomethylating agent; NR, not reported; VEN, venetoclax

**Fig. 1:**
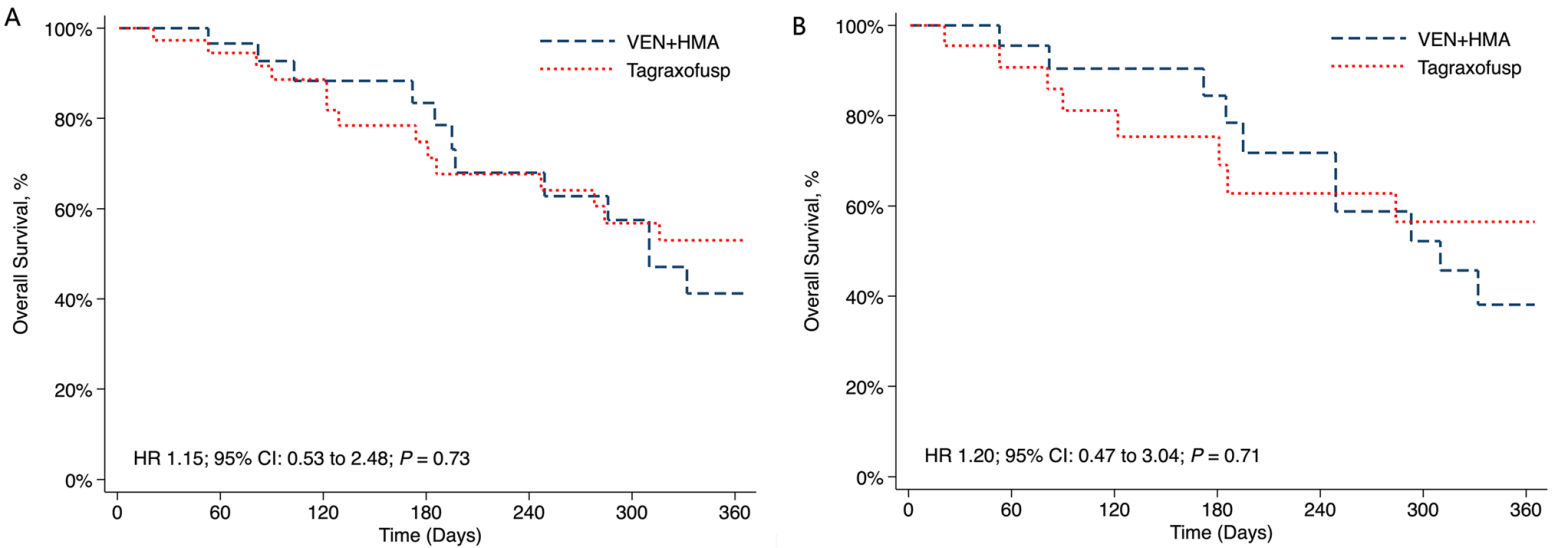
Overall survival at 12-months among newly diagnosed BPDCN patients who received VEN + HMA compared to Tagraxofusp in **A**) patients ≥ 60 years of age and **B**) patients ≥ 75 years of age **A**) **B**) Abbreviations: BPDCN, blastic plasmacytoid dendritic cell neoplasm; HMA, hypomethylating agent; VEN, venetoclax

In this multicenter, retrospective cohort study, we show that the non-intensive regimen of VEN + HMA produced comparable short-term survival outcomes to tagraxofusp for the management of BPDCN. With a median age of 66–70 years at diagnosis, many BPDCN patients are not great candidates for intensive, multiagent cytotoxic chemotherapy. Although tagraxofusp monotherapy offers a more direct targeted approach, demonstrating an impressive overall response rate of 90% with a 12-month OS of 62% in its pivotal trial, significant treatment-related toxicities such as hepatotoxicity and capillary leak syndrome can be prohibitive [8]. Moreover, tagraxofusp or other CD123-targeted agents are not always readily available throughout the world nor are most hospitals equipped to manage the unique constellation of toxicities associated with these therapies.

There are several limitations to acknowledge from this study. First, the data from the TriNetX platform is not curated, and some bias could be present. Second, assessment of prognostic characteristics such as cytogenetics and site involvement (i.e., CNS or lymph nodes) were not available through the TriNetX platform, thus could not be evaluated. Lastly, assessment of treatment response was not feasible via TriNetX given the need to comprehensively consider bone marrow, lymph node, dermatologic, and cerebrospinal fluid findings as well as positron emission tomography/computed tomography imaging to evaluate this endpoint.

In summary, VEN + HMA appears to be a safe and effective therapeutic option in older BPDCN patients not candidates for tagraxofusp or when tagraxofusp is unavailable. Further studies are warranted to prospectively validate these findings.

## Electronic supplementary material

Below is the link to the electronic supplementary material.

Supplementary Material 1

## Data Availability

The data utilized for this study are available from TriNetX and restrictions apply to the availability of the data. Data can be made available at https://www.trinetx.com with the permission of TriNetX.
