# Supplementary material for: Venetoclax and hypomethylating agents versus tagraxofusp in older patients with blastic plasmacytoid dendritic cell neoplasm
Source: Ann Hematol. 2025 Feb 20;104(3):2069–71. doi: 10.1007/s00277-025-06221-4 (PMC12031789; doi:10.1007/s00277-025-06221-4)
Supplement: Supplementary file 1 — Supplementary Material 1 [file 277_2025_6221_MOESM1_ESM.docx]

Supplemental Table 1. ICD-10 Codes for Comorbidities

| **Kidney Disease** | |
| --- | --- |
| ICD-10 N17 | Acute kidney failure |
| ICD-10 N18 | Chronic kidney disease |
| ICD-10 N19 | Unspecified kidney failure |
|  |  |
| **Liver Disease** | |
| ICD-10 K70 | Alcoholic liver disease |
| ICD-10 K71 | Toxic liver disease |
| ICD-10 K72 | Hepatic failure, not elsewhere classified |
| ICD-10 K73 | Chronic hepatitis, not elsewhere classified |
| ICD-10 K74 | Fibrosis and cirrhosis of liver |
| ICD-10 K75 | Other inflammatory liver diseases |
| ICD-10 K76 | Other diseases of the liver |
| ICD-10 K77 | Liver disorders in diseases classified elsewhere |
|  |  |
| **Respiratory Disease** | |
| ICD-10 J00-J06 | Acute upper respiratory infections |
| ICD-10 J09-J18 | Influenza and pneumonia |
| ICD-10 J20-J22 | Other acute lower respiratory infections |
| ICD-10 J30-J39 | Other diseases of upper respiratory tract |
| ICD-10 J40-J4A | Chronic lower respiratory diseases |
| ICD-10 J60-J70 | Lung diseases due to external agents |
| ICD-10 J80-J84 | Other respiratory diseases principally affecting the interstitium |
| ICD-10 J85-J86 | Suppurative and necrotic conditions of the lower respiratory tract |
| ICD-10 J90-J94 | Other diseases of the pleura |
| ICD-10 J95 | Intraoperative and postprocedural complications and disorders  of respiratory system, not elsewhere classified |
| ICD-10 J96-J99 | Other diseases of the respiratory system |
|  |  |
| **Cardiovascular Disease** | |
| ICD-10 I30 | Acute pericarditis |
| ICD-10 I31 | Other diseases of the pericardium |
| ICD-10 I32 | Pericarditis in diseases classified elsewhere |
| ICD-10 I33 | Acute and subacute endocarditis |
| ICD-10 I34 | Nonrheumatic mitral valve disorders |
| ICD-10 I35 | Nonrheumatic aortic valve disorders |
| ICD-10 I36 | Nonrheumatic tricuspid valve disorders |
| ICD-10 I37 | Nonrheumatic pulmonary valve disorders |
| ICD-10 I38 | Endocarditis, valve unspecified |
| ICD-10 I39 | Endocarditis and heart valve disorders in diseases classified elsewhere |
| ICD-10 I40 | Acute myocarditis |
| ICD-10 I41 | Myocarditis in diseases classified elsewhere |
| ICD-10 I42 | Cardiomyopathy |
| ICD-10 I43 | Cardiomyopathy in diseases classified elsewhere |
| ICD-10 I44 | Atrioventricular and left bundle-branch block |
| ICD-10 I45 | Other conduction disorders |
| ICD-10 I46 | Cardiac arrest |
| ICD-10 I47 | Paroxysmal tachycardia |
| ICD-10 I48 | Atrial fibrillation and flutter |
| ICD-10 I49 | Other cardiac arrythmias |
| ICD-10 I50 | Heart Failure |
| ICD-10 I51 | Complications and ill-defined descriptions of heart disease |
| ICD-10 I52 | Other heart disorders in diseases classified elsewhere |
| ICD-10 I5A | Non-ischemic myocardial injury (non-traumatic) |
